# Surgeon stress, anxiety, and workload: a descriptive study of participant reported responses to fundamentals of laparoscopic surgery exercises

**DOI:** 10.1007/s00464-024-11238-3

**Published:** 2024-09-13

**Authors:** Aaron K. Budden, Amanda Henry, Claire E. Wakefield, Jason A. Abbott

**Affiliations:** 1 Discipline of Women’s Health, School of Clinical Medicine, Faculty of Medicine and Health, UNSW, Sydney, Australia; 2 Gynaecological Research and Clinical Evaluation (GRACE), Royal Hospital for Women, Sydney, Australia; 3 Department of Women’s and Children’s Health, St George Hospital, Sydney, Australia; 4 The George Institute for Global Health, UNSW Medicine and Health, Sydney, Australia; 5 Discipline of Paediatrics, School of Clinical Medicine, UNSW Sydney, Sydney, Australia; 6 Behavioural Sciences Unit, Sydney Children’s Hospital, Sydney, Australia; 7 Department of Obstetrics and Gynaecology, Coffs Harbour Hospital, 343 Pacific Highway, Coffs Harbour, 2450 Australia

**Keywords:** Stress, Anxiety, Workload, Laparoscopy, Endoscopy, Surgery

## Abstract

**Background:**

Stress while operating is an important contributor to surgeon health and burnout. Measuring stress is key to improving surgeon and patient outcomes, however biological responses to stress during surgery are variable and difficult to interpret. Participant reported measures of stress have been suggested as an alternative, but the most appropriate measure has not been defined. This study’s primary aim was to assess measures of anxiety, stress, and workload before and after surgical simulation and characterize the relationship between these measures.

**Methods:**

Surgeons completed three laparoscopic exercises from the fundamentals of laparoscopy program (peg transfer, pattern cutting, intracorporeal suturing) in a neutral environment and “stressed” environment (ergonomic, noise, or time pressure). State trait anxiety and self-reported stress on a visual analogue scale were collected prior to simulation and again immediately afterwards. The NASA task load index (TLX) was also administered post-simulation.

**Results:**

Of the 26 participants from gynecological and general surgery specialties, state anxiety increased in 98/148 simulations (62%) with a significant mean increase during simulation (32.9 ± 7.9 vs 39.4 ± 10.2, *p* < .001). Self-reported stress increased in 107/148 simulations (72%), with a significant increase in mean scores during simulation (38.7 ± 22.5 vs 48.9 ± 23.7, *p* < .001). NASA-TLX scores immediately after simulation ranged from 40 to 118 (mean 60.5 ± 28.7). Greater anxiety and stress scores were reported in “stressed” simulations (43.6 ± 23.1 vs 54.2 ± 23.3; 68.7 ± 27.0 vs 52.4 ± 28.2 respectively) with a significant interaction effect of the “stressed” environment and type of exercise. Anxiety and stress were moderately positively correlated prior to simulation (*r* = .40) and strongly positively correlated post-simulation (*r* = .70), however only stress was strongly correlated to workload (*r* = .79).

**Conclusion:**

Stress and anxiety varied by type of laparoscopic exercise and simulation environment. Correlations between anxiety and stress are stronger post-simulation than prior to simulation. Stress, but not anxiety, is highly correlated with workload.

**Graphical Abstract:**

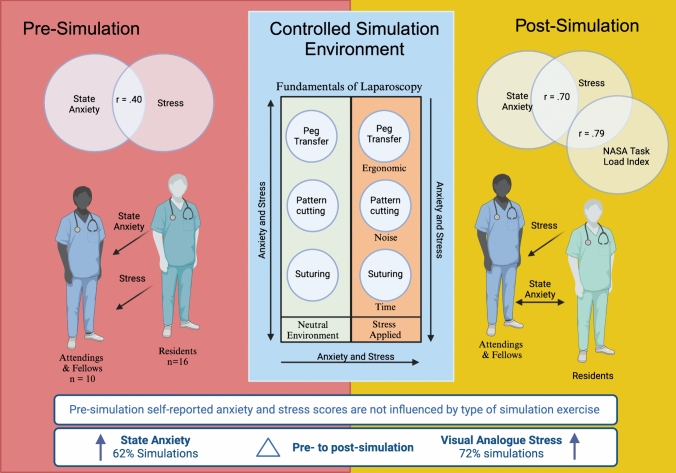

Surgeon stress that occurs while operating is an important contributor to surgeon health and burnout [1, 2] and may affect patient outcomes [3]. Although system-related stressors of surgery (for example high caseload, patient suffering and death, and shift work) may be considered innate to the profession, considerable attention has been directed toward identifying modifiable surgical stressors. These include surgical approach [4], equipment [5], vision [6] and surgeon training [7]. Despite some research into surgeon stress over the past two decades [3, 8], measuring biological response to stress during surgery presents multiple difficulties, with inconsistent changes identified across previous cohorts of surgeons [9].

Cognitive theory suggests that a stressor must be perceived before a reaction to that stressor occurs [10]. Therefore, participant reported measures (PROM) of stress have been investigated as an alternative to biological measures [11], with previous studies incorporating the State Trait Anxiety Inventory (STAI) [12], Dundee State Stress Questionnaire [13], NASA Task Load index (NASA-TLX) [4] and Likert Scales [14] to assess surgeon stress. However, prior PROM literature investigating stress activation has led to confusion by using overlapping descriptions for measures of stress, anxiety, and workload. While stress is broadly considered a cognitive and biological response to a stimulus [15], anxiety is an anticipatory reaction to a stressor that may or may not be present [16] and measures of workload reflect the balance between task demands and an individuals motivated coping capacity across multiple domains whilst undertaking a task [17].

To date, studies are yet to define the most appropriate PROM to assess the impact of surgery on surgeons. Elevations in some biological measures of stress prior to surgery suggest that surgery may provoke anxiety [9] while others have demonstrated a stress response during surgery [18]. Although stress and anxiety have been demonstrated to increase during surgery [19], and can differ between groups based on surgical approach [12], the interaction between anxiety, stress, and workload is unclear. Understanding how these interact is essential in developing a reliable and valid self-reported measure of surgeon stress before the efficacy of stress reduction interventions can be accurately evaluated.

This study measures anxiety, stress, and workload in a well-controlled surgical simulation setting, with defined and reproducible simulations and known stressors. The primary aim was to assess anxiety, stress and workload before and after surgery and then to characterize the relationships between anxiety, stress, and workload in surgeons when conducting laparoscopic simulations. The secondary aims were to assess whether experience, type of simulation, or presence of environmental stressors modify these relationships.

## Materials and methods

A prospective study of surgeons undertaking minimally invasive surgery exercises was conducted at a university teaching hospital in Sydney, Australia. The study hospital has an established and certified training program in minimal access surgery. Ethical approval for this study was granted by the Local Human Research Ethics Committee (No: 17/123) and written informed consent was obtained from participants.

### Participants

Surgeons who were currently employed in a role where they would be performing or training in laparoscopic abdominal or pelvic surgical procedures were invited to participate. Participant data including date of birth, gender, body mass index, level of training (junior trainee, fellow, attending), years of surgical experience (< 5, 5–10, > 10), and number of hours operating each week (< 10, 10–20, > 20) were collected at enrolment. Current medication use was recorded, and participants were required to notify the research team if any new medications were started during the study period.

### Simulation procedures

Participants completed 6 simulations during the 12 months study. Simulations were carried out at the same time of day between 7-10 am, with each exercise demonstrated to the participant on a computer prior to the measured performance. Each exercise was repeated twice, however participants could only undertake one simulation per day. Participants were excluded from participation following morning ingestion of caffeine or when having worked after midnight the night prior.

Simulation exercises were based on the validated fundamentals of laparoscopy (FLS) program [20] however participants were not expected to meet FLS competency rates. The FLS program does not form compulsory part of training in Australia although participants will have been exposed to the types of exercise. We chose to limit the study to three exercises to fit the timeframe and study budget. The ligating loop exercise was removed due to the short timeframe required to complete the exercise and the suturing with extracorporeal knot exercise was removed due to similarity with the intracorporeal knot task. Participants therefore were required to complete the peg transfer, pattern cutting (cutting a single layer circle from double layer cloth), and laparoscopic suturing with an intracorporeal knot exercises. Participants were asked to suture as many knots as possible in 10 min, however the other exercises were not time restricted.

The first of each exercise was undertaken under “neutral” stress conditions where the only stressor was the task itself. The second attempt had an external stressor: (1) poor ergonomics in the peg transfer exercise where candidates completed the task with instrument passing through box trainer port holes furthermost to where they stood such that the participants’ arms were straight with wrists and elbows raised to shoulder height; (2) noise stress for pattern cutting, where participants wore noise cancelling headphones listening to a sound clip with constant variation between 90 and 97 decibels consistent with that reported for change in cognitive performance [21]; and [3] time pressure for laparoscopic suturing where participants were reminded of their achievement in the first exercise and asked to meet that target within 5 minutes. They were reminded of the time left at 30 s intervals and after 2 min were encouraged to speed up to meet their target. Participants were aware an external stressor would be applied, but unaware what the stressor would be until after they completed the pre-simulation PROM and the simulation began.

### Data collection

Each surgeon completed self-reported measures of anxiety and stress prior to and after simulation, as well as reporting on workload after simulation. Participants completed the trait component of the State Trait Anxiety Inventory (STAI) prior to the first simulation session [22] to assess participants’ typical responses to stressors. Pre-simulation PROMS were completed immediately after watching the instructional video for the pending task, but without knowledge of the external stressor that would be applied during the second repetition.

The State Trait Anxiety Inventory (STAI) and NASA Task Load Index (TLX) are summarized in Table 1. The STAI is a validated measure of generalized anxiety that distinguishes the contributions of dispositional anxiety and the transitory experience of anxiety to performance difficulties during testing situations [23]. We chose the STAI-Y2 (trait anxiety) at entry into the study to assess differences between participants and the STAI-Y1 (State anxiety) was recorded prior to and after each simulation exercise. We measured “stress” on a visual analogue scale (VAS) where surgeons were asked to rate their perceived stress on a 100 mm non-graduated line, with the anchors 1 equal to “not at all stressed” and 100 equal to “very stressed”. Mental workload was assessed using the NASA-TLX, where surgeons responded to 6 domain questions including task mental demand, physical demand, temporal demand, performance, effort, and task frustration [24]. The complex administration of the originally described 2-step TLX have led to many researchers dropping the weighting component [25] and we therefore administered and report the raw NASA-TLX score.

**Table 1:** Summary of STAI and NASA-TLX measures

PROM	State-trait anxiety inventory	NASA task load index
Structure	STAI-Y1 (State anxiety) = 20 items STAI-Y2 (Trait anxiety) = 20 items STAI-6 (Short form) = 6 items	NASA-TLX = 2-step scoring; initial responses followed by 15 questions to determine domain dominance and weight applied to domains based on frequency of response NASA-TLX raw = No weighted component
Scale	4-point likert-type response: 1 = Not at all 4 = Very much so	Six individual 20-point Likert scale responses for mental demand, physical demand, temporal demand, performance, effort, and frustration 1 = Low agreement 20 = High agreement
Score	STAI Y1 = 20–80 STAI Y2 = 20–80 STAI 6 = 4–24	Raw TLX = 1–120 Dimension scores = 1–20

*PROM* participant reported outcome measure, *STAI* State Trait Anxiety Inventory, *TLX* Task Load Index

### Statistical analysis

Data were collected and managed using REDCap electronic data capture tools hosted by Research Technology Services at UNSW Sydney. Statistical analysis was carried out using R Studio [26]. Statistical analysis was only conducted at the end of the study period. We calculated correlations between PROMs at baseline, during simulation, and between timepoints; and differences in mean PROM scores from baseline to simulation as well as between neutral and stressful environmental conditions.

The largest known comparison study using PROMs in surgical simulation included 60 medical students undertaking simulation with and without undertaking mental skills training [27]. Based on their STAI results, we calculated that 39 simulations would be required to achieve a power of 80% and a 5% error to detect a 1 point increase in STAI. Our study was open for a year, with a maximum of 26 participants. With six simulations planned per participant, the number of participants was determined to be sufficient to identify differences between type of simulation and presence or absence of a stressor.

Differences in surgeon demographic data and trait anxiety between attendings and residents were assessed with a Student’s *t*-test. Correlations between PROMs were calculated with the Pearson’s rank correlation test. Mean differences in PROMs between FLS tasks were assessed with a paired *t*-test. One-way analysis of variance was conducted to assess the significance of any differences based on type of external environmental modification and surgical experience. Significance was set at *p* ≤ 0.05.

## Results

### Participants

Data were collected between July 2018 and June 2019, with 26 participants recruited from gynecological and general surgery specialties. Table 2 describes the participants’ characteristics. Twenty-four participants completed all three paired exercises (92%). Two residents completed the neutral and “stressed” peg transfer exercise but were rotated to an external training unit before they could undertake the remaining exercises. Their results were included to assess pre-simulation and simulation differences between anxiety and stress but excluded from stratified analyses of types of simulation and external stressors.

**Table 2:** Participant demographics

	Level of training			
	Consultant	Fellow	Resident	*p*
*n*	4	6	16	
Age (mean (SD))	40.5 (5.1)	32.5 (2.0)	29.4 (3.3)	0.001
BMI (mean (SD))	22.6 (3.2)	22.2 (2.2)	24.1 (2.7)	0.296
Gender = Male (%)	50	16.7	37.5	0.514
Years experience (%)				
< 5	25	50	94	0.001
5–10	25	–	6	
> 10	50	50	–	
Weekly operating hours (%)				
< 10	50	83	100	0.18
10–20	50	16.7	–	
Trait (Mean (SD))	27.1(3.9)	31.9(4.1)	32 (6.5)	0.308

*SD* Standard deviation, *BMI* body mass index

### Participant reported measures prior to simulation

Following participant viewing of the planned exercise but prior to undertaking the simulation, state anxiety scores ranged from 20 to 51 with a mean of 32.9 (SD 7.9) and stress scores ranged from 4 to 87 with a mean score of 38.7 (SD 22.5). Compared to residents, attendings were more likely to report lower stress (28.7 ± 14.8 vs 44.0 ± 24.1, *p* < 0.001) and anxiety (30.3 ± 5.9 vs 34.4 ± 8.5, *p* = 0.03). There were no significant differences in pre-simulation anxiety or stress score between any of the six exercises (Table 3), when stratified by type of exercise (Table 4) or in neutral versus stressed simulations.

**Table 3:** Summary of mean PROM scores by simulation

	Peg board neutral	Peg board ergonomic stress	Pattern cutting neutral	Pattern cutting noise	Suturing neutral	Suturing time pressure	*p*
Anxiety pre-simulation (SD)	34.2 (8.2)	32.2 (6.9)	33.5 (7.5)	32.3 (8.7)	33.6 (8.5)	31.9 (8.3)	0.89
Anxiety during simulation (SD)	33.2 (7.6)	43.7 (8.9)	35.2 (9.5)	38.7 (10.0)	40.5 (8.9)	45.5 (11.1)	0.001
Increase in anxiety	− 0.9 (9.3)	11.5 (11.4)	1.7 (8.2)	6.3 (12.9)	6.4 (11.1)	13.1 (12.0)	< 0.001
Pre-simulation stress (SD)	32.9 (19.6)	36.7 (17.4)	35.0 (25.2)	38.4 (24.2)	44.2 (23.1)	45.6 (24.5)	0.28
Simulation stress (SD)	37.7 (21.2)	59.5 (16.5)	39 (26.5)	43.5 (27.2)	54.5 (18.0)	59.2 (22.7)	0.001
Increase in stress	4.8 (19.1)	22.8 (16.8)	4.2 (9.8)	5.1 (14.2)	11.9 (20.0)	12.0 (13.9)	< 0.001
NASA-TLX (total)	45.1 (23.1)	72.2 (19.1)	46.4 (28.1)	57.1 (32.6)	65.9 (28.4)	77.1 (24.4)	< 0.001

*SD* Standard deviation, *NASA-TLX* NASA task load index

**Table 4:** Summary of PROM by simulation type

	Peg board	Pattern cutting	Suturing	*p*
Pre-simulation anxiety (SD)	33.2 (7.55)	32.9 (8.07)	32.7 (8.38)	0.96
Simulation anxiety (SD)	38.5 (9.7)	36.9 (9.8)	43 (10.3)	0.003
Change in anxiety (SD)	5.3 (12.0)	4.0 (11.0)	9.8 (11.9)	0.048
Pre-simulation stress (SD)	34.8 (18.4)	36.7 (24.5)	44.9 (23.6)	0.06
Simulation stress (SD)	48.6 (21.8)	41.3 (26.7)	56.8 (20.4)	0.005
Change in stress (SD)	13.8 (20.0)	4.7 (12.1)	12.0 (17.0)	0.022
NASA-TLX total score (SD)	51.5 (30.8)	58.6 (25.3)	71.0 (27.0)	0.002

*SD* Standard deviation

### Participant reported measures of stress after simulation

State anxiety scores recorded immediately after the simulation ranged from 20 to 61. An increase in anxiety occurred in 98/148 simulations (62%) with a significant increase in mean anxiety during simulation compared to pre-simulation (mean = 39.4 ± 10.2 vs 32.9 ± 7.9, *p* < 0.001). Although attendings’ anxiety scores immediately after simulation were lower than residents (35.9 ± 10.5 vs 41.5 ± 9.7, *p* = 0.001) there was no significant difference in the rise in anxiety scores from baseline between groups (5.2 ± 10.9 vs 7.0 ± 12.4, *p* = 0.34). Stress scores recorded immediately after simulation ranged from 4 to 95. An increase in stress occurred in 107/148 simulations (72%), with a significant increase mean stress during simulation compared to pre-simulation (mean = 48.9 ± 23.7 vs 38.7 ± 22.5, *p* < 0.001). Attendings’ stress scores immediately after simulation were lower than residents (41.9 ± 22.7 vs 52.9 ± 23.8, *p* = 0.007) with lower increase in stress scores compared with baseline (10.8 ± 22.7 vs 18.5 ± 22.5, *p* = 0.04). NASA-TLX scores were recorded immediately after simulation with total scores ranging from 40 to 118 (mean 60.5 ± 28.7).

**Fig. 1:**
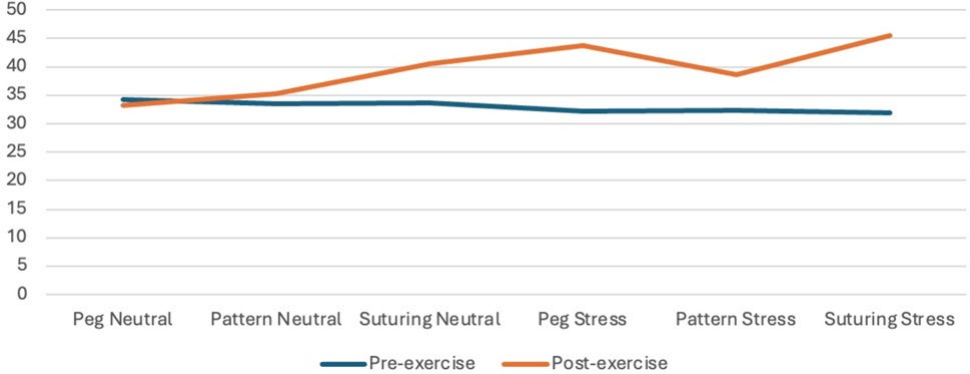
State Trait Anxiety Inventory (STAI-Y1) Score by time of collection

**Fig. 2:**
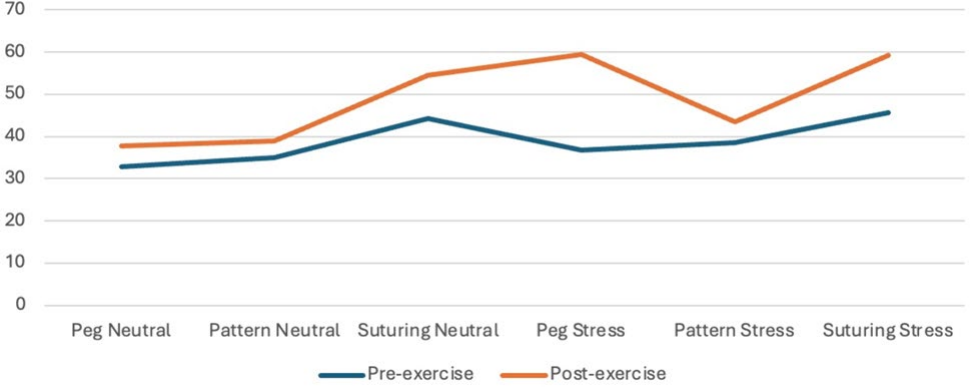
Self-reported visual analogue scale for stress by time of collection

Mean anxiety and stress scores collected after the six simulation exercises demonstrated a significant interaction effect (Table 3, Figs. 1 and 2). Stressed, compared to neutral simulations, were associated with higher post-simulation state anxiety scores (36.2 ± 9.1 vs 42.6 ± 10.3, *p* < 0.001) and self-reported stress (43.6 ± 23.1 vs 54.2 ± 23.3, *p* = 0.001) as well as greater increase from pre-simulation to post-simulation for both state anxiety (2.3 ± 9.9 vs 10.3 ± 12.3, *p* < 0.001) and stress (6.9 ± 17.2 vs 13.6.3 ± 16.6, *p* < 0.01). Intracorporeal suturing exercise was also associated with higher state and anxiety and stress scores compared to peg board and pattern cutting exercises (Table 4).

**Fig. 3:**
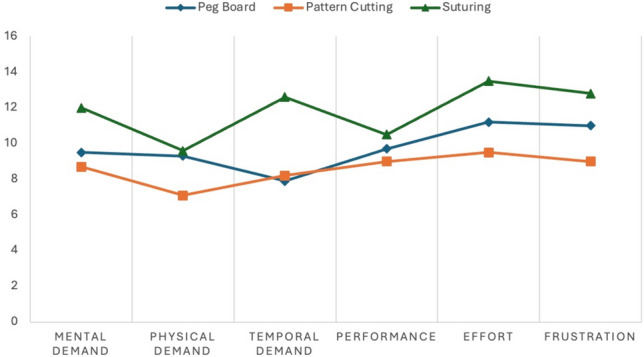
Dimensions of NASA Task Load Index by exercise

Mean NASA-TLX scores stratified by simulation scores ranged from 46.4 to 77.1 and demonstrated a significant interaction (Table 3). Greater total NASA-TLX mean score was reported in the “stressed” simulation (68.7 ± 27.0 vs 52.4 ± 28.2, *p* = 0.001) and in suturing and peg board simulations compared to pattern cutting (Table 4). Except for performance demand, all dimensions of the NASA-TLX were affected by the type of simulation (Table 5, Fig. 3), with higher mean dimensional scores reported in stressed simulations versus neutral simulations.

**Table 5:** Mean dimension scores of NASA task load index by simulation type and presence of a stressor

	Peg board	Pattern cutting	Suturing	*p*	Neutral	Stress	*p*
Mental demand mean (SD)	9.5 (4.6)	8.7 (5.7)	12.0 (5.3)	< .001	9.0 (5.5)	11.1 (5.1)	0.018
Physical demand mean (SD)	9.3 (5.5)	7.1 (4.9)	9.6 (5.3)	.029	6.9 (4.7)	10.5 (5.2)	< 0.001
Temporal demand mean (SD)	7.9 (5.5)	8.2 (5.6)	12.6 (5.0)	< .001	7.9 (5.3)	11.3 (5.7)	< 0.001
Performance demand mean (SD)	9.7 (5.3)	9.0 (5.5)	10.5 (5.7)	.382	8.9 (5.3)	10.7 (5.5)	0.049
Effort mean (SD)	11.2 (5.0)	9.54 (6.1)	13.5 (4.5)	< .001	10.2 (5.8)	12.6 (4.8)	< 0.001
Frustration mean (SD)	11.0 (5.7)	9.0 (6.3)	12.8 (6.0)	.009	9.5 (6.0)	12.5 (5.9)	< 0.001

*SD* standard deviation

### Relationships between stress, anxiety, and workload

Correlations between STAI, VAS stress, and NASA-TLX scores are presented in Table 6. Surgeons’ STAI and stress scores were highly positively correlated prior to, and after simulated exercises. Pre-simulation anxiety scores were weakly positively correlated with post-simulation anxiety, post-simulation stress, and workload. Meanwhile, pre-simulation stress was strongly positively correlated to post-simulation stress and workload. When stratified by type of exercise or neutral versus stress environment, there were no significant differences in the correlations compared to the overall estimate.

**Table 6:** Correlations between STAI, stress VAS, and NASA-TLX scores

	Pre-simulation STAI	Post-stimulation VAS	Post-simulation STAI	NASA-TLX
Pre-simulation VAS	0.40*	0.57	na	0.62*
Post-simulation STAI	0.17*	na	–	na
Post-simulation VAS	0.29*	–	0.70*	0.79*
NASA-TLX	0.21^	–	na	–

*VAS* Visual analogue scale, *STAI* State Trait Anxiety Inventory; *TLX* Task Load Index; *p < .001; ^ = .01; *na* not assessed

## Discussion

To our knowledge, this is the first study to assess the relationships between PROMs of anxiety, stress, and workload in a well-controlled and isolated surgical simulation environment. State anxiety and stress scores were identified to be moderately correlated prior to simulation and strongly correlated post-simulation. Correlations were not significantly different when stratified by type of exercise or by the presence of an external stressful stimulus. Pre- and post-simulation participant reported stress, but not anxiety, was highly correlated with measures of workload. No significant differences were observed in mean State anxiety or VAS for stress prior to simulation between types of laparoscopic exercise or the presence of external stimulus. The strength of correlation between anxiety and stress was greater after the simulation than pre-simulation, with stronger correlations identified when undertaking “stress” simulations and in laparoscopic suturing and peg board exercises compared to pattern cutting. Total NASA-TLX scores similarly were greater in “stress” simulations and in suturing and peg board exercises.

Although pre-surgery and simulation PROMs have been reported, the significance of pre-surgery anxiety and stress with regard to their effects on stress reactions in surgery has received little attention. Previous studies have demonstrated differences in pre-surgery surgeon anxiety, with anxiety scores reported to be higher immediately pre-surgery compared to a non-surgical day baseline [28]; in surgeons-in-training than in specialists [29]; and when acting as primary operator compared with assistants [19]. Although this study similarly demonstrated higher pre-simulation anxiety scores in residents that attendings, there was no difference between groups in the change of anxiety scores from baseline to simulation and therefore changes in anxiety score may not be the most discernable measure of difference in anxiety between study groups. In simulation settings, state anxiety scores have not been shown to differ between types of simulation exercises [30], consistent with the state anxiety scores reported here. Previous studies also report lower state anxiety with repetitive exercise testing [31, 32], however the decreases observed in this study did not reach significance. A particular difficulty in measuring surgeons stress is a lack of clear evidence about the most appropriate tool to use. It is important to consider that anxiety, in contrast to stress, reflects previous events that a participant has been exposed to and may be activated in the absence of a known stressor [33] and different neurobiological response pathways. Therefore, the anxiety scores in the simulation settings may reflect a lack of significant previous neuroendocrine response to stress to create an associated anxiety state to future events [34]. This is an important finding as higher pre-simulation anxiety is likely to affect surgeons’ working memory [35] which in turn can affect procedural performance and stress [36].

Surgeon stress measured by a VAS has been insufficiently researched, with no reports of differences in pre-simulation VAS for surgeon stress between simulation exercises [12, 37, 38]. In contrast, anticipatory stress in live surgery, similar to anxiety, has been reported to be higher in trainees than specialists as well as in primary versus assistant surgeons [19]. In addition, trainees reported greater increase in stress compared to attendings but not anxiety. These findings along with the moderate correlation observed pre-simulation between anxiety and stress in this study questions if pre-simulation factors can cause variation between state anxiety and stress. A previous cohort study in live surgery however identified a significant difference in surgeon reported stress measured by a VAS between two procedures (excision of endometriosis vs hysteroscopic myomectomy) that was not apparent with concurrent measures of state anxiety [19]. Under different surgical conditions, it is therefore possible that if either the potential stressful stimuli were significantly different or the environment was less supported [39], that anxiety and stress may differ between procedures. Currently, there is no clear understanding of how surgery creates stress or an anxiety response, and it is pertinent to reconsider the implications of previously reported “stress” in clinical studies.

The proportion of surgeons who demonstrated an increase in state anxiety was consistent with that previously reported in both live and simulation studies [19, 40], however the relationship between stress and anxiety measured during the simulation has not previously been reported. Anxiety and stress measures demonstrated stronger correlation after the procedure and in contrast to pre-simulation, mean stress and anxiety scores after simulation were affected by type of simulation and the presence of a stressor. The underlying reasons for these observed stronger correlations at this time point are not yet clear. Stress would normally be reported in the context of a threat stimulus and the requirement to mount a mental or physiological response [41] to that stress, while anxiety scores typically increase with uncertainty [33]. Anxiety is however also linked to fear, which is the emotional manifestation of stress that occurs in situations where the perception of threat is ongoing [16]. The correlations observed between State anxiety and stress therefore may represent the symbiotic nature of these responses in a dynamic and uncertain procedural environment [35]. This hypothesis is supported by the absence of a difference in the anxiety-stress relationship between neutral and stress simulations as the external stressor was consistent and would not have introduced additional uncertainty into the simulation. The different types of exercises however, were unique in the procedural elements and may explain the differences in the anxiety-stress relationship observed.

Stress and mental workload are two related concepts that originate from different theoretical frameworks [42], with some authors arguing that high workloads can exist independent of stress [36]. Despite the strong correlations observed between anxiety and stress post-simulation it was only stress, both pre-simulation and post, that had a strong relationship to workload. It has been argued that stress is an important component of working memory and mental workload to be able to complete a task [43], and while strong positive correlations may be expected between procedural stress and workload, it remains unclear how pre-simulation stress is strongly related to procedural workload.

Strengths of this study include a well-defined study protocol, the use of well-defined and repetitive laparoscopic simulation exercises, standardized stressful environmental stimuli defined by the literature, and conduct of the study in isolation to the work environment. Limitations of the study include small number of participants and the potential for the included simulation to fail to provoke an anxiety response due the isolated study design. Also, although the type of stressors used in this study have been well described, the degree/severity and exposure time of those stressors needed to elicit a response have largely not been specified and may not have been sufficient to evoke a response in all participants [44]. Future studies could consider expanding participant numbers as well as consider the demographics of those participants and societal expectations may influence self-reported anxiety and stress. In addition, future studies should consider controlled introduction of potential stress confounders that commonly occur in surgeon work life such as caffeine ingestion and sleep disturbance.

Anxiety, stress, and mental workload have been shown here to be associated with undertaking simulations. Although pre-surgical measurement of anxiety may have value in the live surgical setting, pre-exercise measurement of anxiety in highly isolated simulations was of limited value. Stress scores were highly correlated with the total NASA-TLX score however collection of NASA-TLX dimension scores also allowed observation of which simulations affect different dimensions. The combination of measuring stress and mental workload may provide a greater understanding of the impact of surgery on surgeon.

## Conclusion

Stress and anxiety as measured by STAI are highly positively correlated after surgical simulation, but less strongly correlated prior to simulation. Stress, both prior to simulation and after simulation are highly correlated to overall workload scores. Stress and anxiety scores post-simulation fluctuate based on type of simulation exercise and external environment. The narrow variation in pre-simulation in anxiety or stress scores may reflect the isolated study environment and further work in the live setting is required to identify pre-surgical differences of anxiety and stress to further understand their role in assessing the impact of surgery on surgeons.
